# Collectanea

**Published:** 1832-09

**Authors:** 


### COLLECTANEA.

Floriferis ut apes in saltibus omnia libant,
Omnia nos, itidem, depascimur aurea dicta.

### PATHOLOGY.

*Remarkable Epidemic.* THE effects of epidemia are infinitely varied. I remember, at Oxford, some years ago, seeing a case of an epidemic which consisted in the mortification of the thumb to the first joint, and attacked nearly a whole village. * * The pestilence which began about A.D. 169 was characterised by a loathsome gangrene of the feet, probably similar to the mortification of the thumb which I saw in Oxfordshire.—*Forster on Cholera Morbus and Epidemic Diseases.*

### *Furious Delirium, following Revulsion of Erysipelas of the Thigh, cured by the Return of the Inflammation.*

A MAN, forty-five years of age, was wounded, on the 25th of last November, by a pointed instrument, which penetrated the thigh about four inches in depth, and grazed, but without wounding, the crural artery. M. BLONDIN found him in the following state: face red; tongue whitish in the middle, red at the edges; pulse one hundred per minute; headach; and a little redness and pain in the neighbourhood of the wound. He was bled, and put on the antiphlogistic regimen. On the following day, (for M. Blondin had not been able to see him the over-night,) the state of the patient was very alarming: the countenance was flushed, the pulse 130 per minute; look wild; intellect lost; delirium furious; motions violent, &c. On inquiring of the attendants as to what had occurred since the last visit, M. B. learned that the thigh, just round the wound, had assumed a purplish red hue, some hours after his departure; that there had been much heat and pain, and that there had been applied compresses steeped in very cold vinegar and water; under the influence of which the heat and redness, and even the pain also, had entirely disappeared; and that there only remained a slight yellowish discoloration of the skin, which tint was very perceptible; and, lastly, that the cerebral symptoms had immediately (as if metastatically) manifested themselves with extreme violence.

M. Blondin believing that there was in this case a metastasis to the brain or its membranes, bled the patient largely from the arm, applied twelve leeches behind the ears, put sinapisms to the feet, and ordered a composing draught and a fever mixture.

On the fourth day a little amendment was perceived; and then he was bled again, leeches reapplied, a purgative enema was administered, and frictions with tartar-emetic ointment ordered to the parts which the erysipelas had formerly occupied.

On the fifth day, the inflammation of the skin reappeared, and spread over all the upper and interior third of the limb; the cerebral symptoms disappeared, and the patient only complained of slight headach and debility. The following day the erysipelas continued spreading, and was accompanied with fever, &c. It was combated with frictions of mercurial ointment, and at the end of eight days the disease was completely subdued."—*Lancette Française.*

### PRACTICAL MEDICINE.

*Tic Douloureux cured by Stramonium,* by Dr. FOTT.—A young lady had suffered for years from attacks of this distressing malady: the paroxysms terminated by a swelling of the face and lips on the side affected. Various remedies had been tried without avail. Dr. Fott cured her in six weeks, by making an issue in the arm, and by giving her fifteen drops of the tincture of stramonium every three hours.—*Beitrage Mecklenbergtschen, Aerzte* 1832.

### *Effects of Croton (Tiglium) Oil, employed externally and internally, as observed by* Professor Andral.

For some time past M. Andral has been engaged in making many trials of the oil of croton tiglium, administered both internally and used externally. The following are the results of his observations on the effects of this potent medicine.

A drop of the oil of croton tiglium, exhibited in pills or in a teaspoonful of syrup or of ptisan, to a person whose stomach is in a healthy state, produces immediately a sensation of burning in the mouth, in the fauces, and sometimes even along the whole course of the œsophagus; an unusual heat in the stomach, occasionally nausea, but rarely vomiting: this heat passes away in a few minutes. It is an hour or an hour and a half after taking the oil before the first evacuations occur, preceded by flatulent discharges and slight colicky pains, but without tenesmus and without anal heat. The stools are very liquid, come suddenly away, and look sometimes like clear water, and sometimes have a slight yellowish hue; commonly there are eight or ten evacuations in twenty-four hours. On the following day the effects are over; the tongue has its natural appearance; there is a little thirst; the abdomen is soft, but the bowels not open. M. Constant, who has repeated these experiments, never but once met with pains in the stomach which required to be treated by antiphlogistic means.

Most frequently the pulse, observed with care, diminishes in frequency under the action of this medicine; sometimes it does not vary; twice only has it been found to be accelerated; the skin retains its warmth; sometimes there is perspiration noticed, followed by tranquil sleep. As to the urine, it was increased only in one paralytic subject, who, after having taken a drop of the oil, had no other evacuation. Furthermore, administered from half a drop to three drops, it has never occasioned any inflammation of the stomach or intestines.

Applied externally upon some spot of the skin, the oil of croton produces a slight smarting, and some hours after an eruption of a number of small red pimples, which become pustules, having much the appearance of variolous pustules, or of those produced by tartar-emetic ointment.

Four or five drops applied to an equal surface on the palm of the hand, cause a confluent eruption, of which some pustules are surrounded by inflamed areolæ, and occasion acute pain, which, however, passes away in four and twenty hours.

These experiments have been made on more than thirty patients, and the frictions instituted on the abdomen, in the armpits, and on the thighs, with from twelve to twenty drops, pure, or mixed with oil of sweet almonds, in the proportion of from ten to twenty drops of croton oil to one ounce of the oil of sweet almonds. Once there was observed to be three abundant evacuations following the frictions in a few hours. The progress of the eruption is as follows: at the end of from thirty to fifty hours all the pustules are developed, some of them confluent, and then there are seen large bladders filled with a whitish opaque liquid. The pustules continue to increase in size during three or four days, and then remain stationary; a little afterwards they dry, like the eruption of smallpox. If the spots have been numerous, the skin becomes covered with scabs, which desquamate slowly.

If the pustules are few and remain small, M. Andral says their desiccation takes place without the formation of any scabs. Generally, the whole disturbance ceases about the eighth or eleventh day. Once only the eruption wholly failed being excited; but it varies in intensity according to the number of drops of the oil employed, and according to the particular sensibility of individuals and the regions of the body submitted to these frictions. Five or six drops have sufficed in most cases to bring out a considerable eruption, but of small extent; with twelve or fifteen drops a confluent eruption may be produced, covering a great part of the abdominal parietes. In these experiments the number of drops has never exceeded twenty. On the skin of the face the eruption has always been considerable, both as respects the size and the number of the pustules.

At the close of this summary, "La Lancette Française" reports many other cases in which the croton oil has been employed by M. Andral both externally and internally, and with complete success. These have been abridged in the "Archives générales," and we shall make some extracts from the abridgment.

*External use.* A man, aged fifty-four, who some time previously had erysipelas of the face and scalp, accompanied with adynamic symptoms, was admitted into La Pitié, towards the end of last October, with paralysis of the left side of the face: the affection was characterized by insensibility of the parts, and almost total loss of sight, hearing, taste, and smell, still without there being any distortion or paralytic motion of the mouth. He was bled and purged, without benefit; but eight drops of croton oil being rubbed on the affected side of the face, caused a confluent eruption, and at the end of two days all the symptoms had disappeared, and the cure was complete.

A painter, aged forty years, who had undergone seven mercurial courses, was seized, after a sexual debauch, with a paralytic affection of the muscles of the lip and of the right cheek, with numbness of all that side of the face, but without loss of feeling. A friction with eight drops of oil of croton was followed with a similar result to the preceding case, and in a few days the cure was complete.

A joiner, fifty years old, who had suffered with sciatica for about twenty years, which, although relieved often by oil of turpentine, still continually recurred, after one friction with the oil of croton was entirely cured in less than three days.

A labourer, forty-eight years of age, affected with a like ailment, which had lasted for four months, and which extended from the hip to the sole of the foot, used thirty-two drops of croton oil in four frictions, made at intervals of a single day, on the posterior part of the lower extremity, from above downwards. A very painful eruption followed these frictions, but the sciatic pains diminished even by the first friction, and disappeared, as if by enchantment, after the fourth.

Similar benefits were received by a woman, fifty-eight years old, and who was afflicted with a similar disease.

In two cases of chronic affection of the stomach, which had resisted ordinary treatment, frictions with oil of croton, having produced confluent eruptions, acted like a powerful counter-stimulant, and restored the appetite, removed the pains of the stomach, restrained the vomitings, and so forth.

Other instances are reported, but, as they are less interesting than those already above recorded, we shall pass them by, to relate some cases of the benefits derived from its *internal use.*

A shoemaker, aged thirty-five, of a tolerably good constitution, was admitted into the hospital affected with laryngitis and chronic bronchitis, complicated with violent asthmatic symptoms. Bleeding and demulcent drinks gave no relief. M. Andral then administered a drop of croton oil in a teaspoonful of ptisan: this was followed by vomiting and five abundant stools. From this moment the improvement was obvious; but, as the larynx appeared to be the principal seat of the evil, the front part of the throat was rubbed with ten drops of the same oil, which caused a confluent eruption of pustules. The respiration became more free; the voice, at first scarcely audible, became much more clear: in short, after the application of twenty leeches to the upper part of the sternum and the exhibition of four grains of calomel, the condition of the patient was very satisfactory.

In another case of chronic bronchitis, with incipient hypertrophy of the heart and very frequent attacks of asthma, which for some years past had tormented a "limonadier," aged twenty-five years, the internal administration of a drop of the oil of croton produced many evacuations, and was followed by very notable relief from his sufferings.

A woman, aged fifty-five, had suffered for a month with vomiting both of solid and liquid food: she attributed this disorder to a journey of sixty leagues that she had performed in a carriage which was badly hung, and in which she was violently jolted. There existed at the same time a prolonged constipation, which the patient declared had lasted for a month. Her emaciated form expressed anxiety and suffering; her countenance was pale, and pulse slow. On examination, two irregular moveable tumors were observed, one seated in the right hypochondrium, the other towards the middle of the abdomen, a little above the umbilicus. She was put upon soothing treatment, with a milk diet; and at the same time, to overcome the costiveness, two pills were given, each containing two grains of the resin of jalap and two grains of aloes. This dose produced no further effect than some flatulent eructations and slight colicky pains; no stools followed. The dose of jalap was gradually increased to eight and even to sixteen grains, without causing any evacuations: at last, thirty-two grains of the jalap resin and a drop of croton oil being taken without effect, three drops of the oil were exhibited, which produced five liquid evacuations containing scybalæ. This treatment slightly relieved the patient; and M. Andral having discovered a crural hernia, which she said she had had for many years, transferred her to M. Velpeau, who reduced the hernia by the use of belladonna frictions to the tumor, and introducing into the rectum pledgets of cotton impregnated with the same substance; but the vomiting still continued, the emaciation increased, the costiveness became again as obstinate as before the administration of the croton oil, and the patient died under the care of M. Andral, in the last stage of marasmus. On examining the body after death, the tumors, the existence of which was detected during life, were found to be, the one the gallbladder containing a calculus as large as a pigeon's egg, the other a schirrous mass formed by the cohesion of the stomach, transverse arch of the colon, and the cœcum. The caliber of the colon was so much contracted that it was with difficulty the ringfinger could be introduced.

A shoemaker, twenty-three years old, was tormented for a month with an obstinate headach, attended with noises in the ears, vertigoes, and indistinctness of vision. Bleeding and mustard pediluvia afforded very slight relief, but a drop of croton oil taken two hours after venesection, and which produced twenty liquid evacuations from the bowels, effected by the next day a perfect cure.

A young man, about thirty years of age, lying No. 9, St. Leon's ward, had suffered for eight years with the most violent headachs. His sight had been progressively getting weaker, and the pupils were in a constant state of dilatation. His right arm was affected with tingling sensations; the lower limbs were numbed; and from time to time he was seized with convulsions and delirium. He had been bled both generally and locally, a seton had been made in his neck, but nothing afforded him any relief; when at last he tried the croton oil, which was administered in pills every two or three days, in doses of a quarter, a half, and an entire grain. Diarrhœa for several days succeeded the costiveness which was habitual to him. Under the influence of this treatment his condition was so much improved that, at the end of a few weeks, he left the hospital entirely cured.

A hosier, aged thirty-two, admitted into the hospital on the 3d of November, presented the following symptoms, which by authors have been said to distinguish asthma: he had intense dyspnœa, respiration accompanied with a very decided wheezing and sonorous rattling; percussion on the chest gave a tympanitic sound, and yet auscultation could not detect more than a weak respiratory murmur, thus proving that the air did not enter the pulmonic cellules. He was bled largely, but without benefit, on the day of his admission; a drop of the croton oil was exhibited, which procured many abundant stools. On the next day, the rattling in the throat had considerably lessened, the respiration was more free, and the patient was delighted with his condition, which became still more improved by another purgation two days afterwards, with a pill containing a quarter of a grain of the oil. All things from this time went on well, and in a few days the man went out cured.

Furthermore, M. Andral has administered the medicine, the effects of which have been now detailed, in five severe cases of painter's colic, with the greatest success: two or three days sufficed to effect the cures of all these individuals. —*La Lancette Française.*

---

*On the Cure of Amenorrhœa by Leeches applied to the Mammæ.* By Charles Loudon, M.D., Physician to the Leamington Spa Bathing Institution.

There are but few of the sympathies which exist between the remote parts of the body which so decidedly manifest themselves as that between the uterus and mammæ. It would, therefore, be useless to point out how many physiological and pathological facts demonstrate this in practice. The father of medicine was not ignorant of this great sympathy, and availed himself of it therapeutically; for in floodings he recommends dry cupping to be practised on the breasts, with the view, no doubt, of causing a revulsion, and exciting a new action in the womb.

Reflecting on this principle, it occurred that, if an action could be induced in the capillary vessels of the mammæ, the womb might in other diseases be made to sympathise with these parts. Leeches seemed to be the most likely means of producing this action, and in a case of amenorrhœa of two years standing, two leeches were applied to the lower part of each breast for a month, repeating them on alternate days. In three weeks the mammæ swelled to an enormous size, giving a sensation to the patient as if they would burst. About the end of the month menstruation came on, and the young lady is now the mother of two children. Several other cases in which the leeches have been tried, have been followed by the same results, and no medicine has been used excepting an aperient to keep the bowels open.

Although this remedy is submitted to the profession as a very certain means of exciting uterine action in this disease, and is founded both on the principles of physiology and pathology, it is not held up as a specific in all cases of amenorrhœa, and is not intended to supersede, but to be combined with the other auxiliaries in the treatment of that disease. Hence purgatives, local and general, vapour baths, hellebore, and the other remedies which experience has pointed out as useful, should not be neglected. Nor is the author of the present notice aware that this practice did not exist at some previous time in the history of medicine, although, if it did exist, on what grounds it was abandoned, it is difficult to conceive. Leeches were used in medicine long before the Christian era; mention being made of them both by Pliny and Galen.—*Edinburgh Med. and Surg. Journal.*

---

### SURGERY.

*Case of Chronic Hydrocephalus successfully treated by Puncture.* By R. C. Russel, Esq., Surgeon, Gallowgate Head, Aberdeen.

Christian Littlejohn, whose age is eight months, was affected with chronic hydrocephalus. Her mother observed, a few days after birth, a greater separation of the bones of the head than natural, after which its size began to increase very rapidly. Eleven weeks after birth, I was requested to see her along with my friend Mr. Moir, lecturer on anatomy in this place. By that time the head had acquired an enormous size; it measured in circumference twenty-three inches, and from the meatus of one side to that of the other, across the vertex, fifteen and a half inches. There was a constant rolling of the eyes and squinting, but there was no unusual dilatation of the pupil, which contracted readily on the application of light. The bowels were irregular, and she was affected with slight startings during sleep. Various methods of treatment had been adopted, viz. compression, blisters, mercury, diuretics, &c.; but, in spite of these measures, the head continued to increase. As the general state of her health appeared good, I resolved upon trying the operation which has been recommended, of gradually discharging the water by puncture. The operation was accordingly performed on the 25th August, six days after my first visit. The instrument which I employed was a trocar such as is used in hydrocele. I introduced it about half an inch in depth on the right side of the anterior fontanelle, and three ounces of serous fluid were discharged through the cannula. A piece of adhesive plaster was placed over the puncture, and a roller applied around the head. She slept well that night, but next day she was slightly feverish, and continued so for two days afterwards, when she appeared as well as before the operation.

On the 4th day of September, the puncture was repeated in the same manner on the opposite side, and five and a half ounces of turbid serum were evacuated, containing several flakes of lymph. No unfavorable symptom followed. On the 15th September, the size of the head appeared much lessened, and was found to have diminished two inches and a half in circumference, and two and a quarter across the vertex. Ossification had made considerable progress. A large opening in the frontal bone, which extended from the bregma to the nose, was completely filled up, while those in other parts were much diminished. In again using the trocar, only an ounce of fluid was discharged. On the 5th of October, I inserted the trocar near to the part I first punctured, and introduced it as far as the meninges, but only a half ounce of fluid passed through the cannula; I therefore reintroduced it, and entered it obliquely, about an inch and a half in the direction of the ventricle, and upon withdrawing it, nine ounces of serum were discharged in a continued stream. The wound was closed, and a roller applied tightly around the head. Immediately after the water was discharged, the pulse became feeble, and she was faint and weak, but during the evening she fell asleep, and awoke an hour afterwards apparently much refreshed. To my great surprise not one unfavorable symptom followed. The pulse indeed became more regular than it had hitherto been, the startings during sleep were not so frequent, and she appeared in other respects better, with the exception of her bowels, which continued to discharge stools of a dark green colour. She continued to improve for nearly three weeks afterwards, when her former symptoms gradually returned, and an obscure fluctuation could be perceived by pressing with the fingers above the anterior bregma. Small doses of calomel were administered till the mouth was affected, which shortly produced an absorption of the fluid, and a removal of all the hydrocephalic symptoms. Since then, she has had no relapse, and has enjoyed almost uninterrupted good health. She is a stout and lusty child, and her size uncommonly large for her age. The bones of her head are now complete, excepting the anterior opening, which is closing. The size of the head is less by four inches in circumference, and two and a half across the vertex than it was previously to the first operation. With the exception of Dr. Conquest's two cases,* I am not acquainted with another in which the ventricle has been punctured for the relief of water in the head. In the cases of Rossi, and Dr. Vose, the water between the membranes only was evacuated. An opinion is entertained by several, that this operation is not only a very dangerous but an extremely doubtful one. I trust, however, that the result of these

* Vide London Med. and Phys. Journal, for May 1830, p. 415; and December 1830, p. 515.—Editors.

cases will prove that such fears are in a great measure groundless, and that, under favorable circumstances, the chance of cure is such as to justify its performance.—*Edinburgh Med. and Surg. Journal.*

---

MISCELLANEOUS.

*On the Employment of Chloride of Lime to destroy the Smell of Paint.* The odour arising from painting being highly disagreeable to many persons, medical men are frequently consulted as to the most effectual means of removing the smell: for this purpose the chloride of lime (chloruret of calcium,) appears completely to succeed. The following is the method most conveniently pursued.

Boards three feet long, and two wide, should be arranged in the rooms to be submitted to the influence of the chlorine, and hay loosely scattered upon them. The dry chloride should be well dusted over the hay, and left for several days; the apartments during the time being shut up. Chlorine is liberated from the chloruret by the decomposing power of the carbonic acid of the atmosphere, and pervading the chambers, annihilates the smell of the painting.

If it is wished to remedy dampness, as well as to remove smell, it is advisable to put into plates or upon trenchers, pieces of muriate of lime, which, being deliquescent, absorbs moisture from the atmosphere, and melts. Some persons have proposed to put pieces of quick lime on the ground at the same time that the chloruret is being used. This plan is, however, bad; for, under ordinary circumstances, the chloruret is not decomposed, unless carbonic acid be present; and the quick lime not only absorbs the moisture, but appropriates at once the carbonic acid which should have decomposed the chloruret and set the chlorine at liberty, which must be regarded as the only agent that gives efficaciousness to the chlorurets. A few small fragments of chloride of lime are likewise found very effectual in removing the unpleasant smell of urine; e. g. after eating asparagus, cabbages, and cauliflowers. Vinegar has been recommended for this purpose, but it only removes the one odour to replace it by another still more disagreeable: and furthermore, six, or eight, or ten, drops of the solution of the chloride are far more effectual than four ounces of acetic acid. The chloride is likewise the best means of removing any unpleasant odour from night tables, and other conveniences of that sort, which are so disagreeable in sick chambers.—(*Bulletin des Sciences Medicales.*)

---

*Extracts from "some Account of the Famine in Guzerat in the Years* 1812 *and* 1813. By Captain James Rivett Carnac, Political Resident at the Court of Guicawar. In a Letter to William Erskine, Esq."

It has often been remarked, that the appearance of locusts is a prognostic of other evils. Flights of these destructive insects first appeared from the eastward in the Bengal provinces, about the beginning of the year 1810, and taking their course in a northerly direction, passed through parts of the country designated by the southern people Hindoostan; and, in the revolution of fifteen months, arrived at the province of Marwar, skirting the large western desert of India. In the year 1811, the annual fall of rain failed in Marwar; and when every vestige of vegetation had disappeared, the locusts made way into the northwest district of Guzerat, named Puttum, and from thence scoured Kattiwar; on one occasion only, appearing as far south as the city of Baroach on the Nerbudda. Beyond this point the locusts were not known to extend; and by the commencement of the monsoon of 1812, this plague vanished from the face of the country.

The destruction committed by these insects in the western parts of Guzerat was deplorable. During the circuit of the subsidiary force at the latter end of 1811, extensive tracts were covered with cultivation; and, until examined, the spectator would have considered the harvest as being in a most flourishing condition. The locusts, however, had devoured the grain, and the stalks were left as unworthy of being cleared from the ground. The failure of grain in Marwar, and the ruin by the locusts of the products of the land during the preceding year, drove the inhabitants of that unfortunate country into the bosom of Guzerat, where their condition was comparatively improved, though one of the causes which compelled them to seek refuge at a distance from home had begun to operate also in that province. Miseries seemed to follow the footsteps of the Marwarees, and to mingle their neighbours in their untoward destiny; for it was in the year 1812 that Guzerat also experienced a failure of rain, when the demands on its resources had augmented in a twofold degree.

The mortality which ensued among the emigrants, who had sought refuge after the sufferings of a famine in their own country, covered with disease, regardless of every consideration but that promoted by the calls of hunger, almost surpasses my own belief, though an unhappy witness of such horrid events.

In the vicinity of every large town, you perceived suburbs surrounded by these creatures. Their residence was usually taken up on the main roads under the cover of trees; men, women, and children, promiscuously scattered, some furnished with a scanty covering, others almost reduced to a state of nudity, while, at the same moment, the spectator witnessed, within the range of his own observation, the famished looks of a fellow-creature, aggravated by the pain of sickness; the desponding cries of the multitude, mingled with the thoughtless playfulness of children, and the unavailing struggles of the infant to draw sustenance from the exhausted breasts of its parent. To consummate this scene of human misery, a lifeless corpse was at intervals brought to notice by the bewailings of a near relative; its immediate neighbourhood displaying the impatience and wildness excited in the fortunate few who had obtained a pittance of grain, and were devouring it with desperate satisfaction. The hourly recurrence of miseries had familiarised the minds of these poor people, as well as of people in general, to every extremity which nature could inflict: in a short time, these emanations of individual feeling among themselves, which distinguished the first commencement of their sufferings, gradually abated, and the utmost indifference universally predominated. I shall venture to give you a few examples, which came under my own eyes, and which, in spite of the painful sensation which they excite, I bring myself to describe, from the desire of elucidating the depression to which a rational being can be reduced.

During the progress of these miseries, I have seen a few Marwarees sitting in a cluster, denying a little water to sustain her drooping spirits, to a woman stretched beside them, with a dead infant reposing on her breast. In a few hours this woman had also expired, and her dead body, as well as that of the child, remained close by them, situated as before described, without a single attempt to remove them, until the government peons had performed that office. I have seen a child, not quite dead, torn away by a pack of dogs from its mother, who was unable to speak or move, but lay with anxious eyes directed to the object of its fond affection. It was pursued by its former little play-mates, which had shared in its extreme adversity; but the ravenous animals (who had acquired an extraordinary degree of ferocity from before having fed on human bodies) turned upon the innocents, and displayed their mouths and teeth discoloured with the remains of the child; a rescue was attempted by ourselves, but the remains of life had been destroyed, and in struggling for its limbs, the dogs had actually carried off one of its arms. I have witnessed those animals watching the famished creatures, who were verging on the point of dissolution, to feast on their bodies; and this spectacle was repeated every successive day in the environs of this town. Lastly, to my knowledge, those feeling and prejudices "concentrating all their precious beams of sacred influence," those which life in ease and affluence would only have resigned with itself, in the extremes of distress, seemed to have lost their power. Distinctions of caste were preserved until the moment when the hand of adversity bore heavy; then the Brahmin sold his wife, his child, sister, and connexions, for the trifle of two or three rupees, to such as would receive them. With these individual cases I will leave you to estimate the extent of mortality; but it is in my power to state as a fact, that the number of the Marwarees who died in a single day at Baroda, could scarcely be counted, and the return of burials in twenty-four hours often exceeded 500 bodies.—*Edinburgh New Philosophical Journal.*

---

*Vegetable Chemistry.* Captain CHARLEP LE HUNTE gives the following as the result of his analysis of the stony pericarp of the *Lithospermum officinale*, one of the most remarkable substances in the vegetable kingdom; its properties, mechanical and chemical, being those of a mineral rather than of a vegetable. The seeds resemble small pear-shaped porcelain beads; they are very hard, difficult to break, and have a high polish. When heated, they at first become black; but they do not shrink, nor does a white heat change their form in the slightest degree; it destroys, however, their lustre, and renders them, when the vegetable matter has been consumed, whiter than they were originally. Before the blowpipe small pointed fragments of the pericarp may be partially fused; but this requires a good heat.

| | |
|---|---|
| *Result of repeated analyses*: Carbonate of lime .............. | 43.70 |
| Silica.......................................................... | 16. 5 |
| Vegetable matter: small quantity of phosphate of lime and oxide of iron, with traces of potash and magnesia........................ | 39. 8 |
| —*Jameson's Journal.* | 100 |

---

